# The Effect of TiO_2_ on the Dielectric Performance of ZrO_2_ and Nb_2_O_5_ Pre-Doped CaCu_3_Ti_4_O_12_ Ceramics

**DOI:** 10.3390/ma17235824

**Published:** 2024-11-27

**Authors:** Guoyu Zhang, Lu Li, Yanxin Nan, Peng Li, Tao Deng, Zhipeng Lei, Yuanyuan Li, Jianhua Zhang

**Affiliations:** Shanxi Key Laboratory of Mining Electrical Equipment and Intelligent Control, National & Provincial Joint Engineering Laboratory of Mining Intelligent Electrical Apparatus Technology, College of Electrical and Power Engineering, Taiyuan University of Technology, No. 79 Yingze West Avenue, Wanbailin District, Taiyuan 030024, Chinaleizhipeng@163.com (Z.L.);

**Keywords:** CaCu_3_Ti_4_O_12_-based ceramics, dielectric properties, temperature stability, good manufacturability

## Abstract

In this work, the effects of different sintering temperatures and TiO_2_ concentrations on the dielectric properties of ZrO_2_ and Nb_2_O_5_ pre-doped CaCu_3_Ti_4_O_12_ (CCTO) ceramics were investigated. These doped ceramics were sintered at temperatures of 1020, 1030, and 1040 °C, exhibiting the lowest dielectric loss of 0.01, which consistently remained below 0.03 across a wide frequency range from 10 to 10^5^ Hz. Simultaneously, they maintained a high dielectric constant of more than 3000 and remarkably met the requirements for an X8P capacitor (Δε′ ≤ ±10% at temperature range from −55 to 150 °C). It was clarified that TiO_2_ doping increased grain boundary resistance, leading to the reduced dielectric loss, and elevated the grain boundary activation energy, thereby enhancing the temperature stability. The TiO_2_-doped CCTO-based ceramics also demonstrated reduced sensitivity to variations in sintering temperature, highlighting their excellent manufacturability. This characteristic holds great promise for the fabrication of CCTO-based ceramics, which usually exhibit poor repetitiveness.

## 1. Introduction

In recent years, electrical equipment with good comprehensive performance has become an important development goal. Dielectric capacitors are essential circuit components that require low dielectric loss (tan*δ*) and good temperature stability. CaCu_3_Ti_4_O_12_ (CCTO) with an extremely high dielectric constant (*ε*′) (>10,000) over a wide frequency range and temperature range has shown great potential as a capacitor-used material [1,2,3,4,5] and has garnered extensive attention and research. However, pure CCTO without a dopant presents a relatively large tan*δ* and limited temperature stability. Moreover, it exhibits high sensitivity to sintering temperature and composition; CCTO samples sintered at the same temperature or within a narrow temperature range, or with a small variation in composition, demonstrate distinct differences in dielectric performance, which is extremely unfavorable to their future manufacturability [6]. In order to achieve excellent dielectric performance and good manufacturability, it is imperative to solve these problems.

Generally, the low tan*δ* in CCTO ceramics is mainly correlated with high grain boundary resistance (*R*_gb_) [7,8,9,10,11,12,13,14,15], which can be effectively tailored through element doping [7,8,10,16,17,18,19,20,21,22,23,24,25,26,27,28,29], such as with Zn^2+^, Zr^4+^, (Sr^2+^, Ni^2+^), (Zr^4+^, Nb^5+^), and (Al^3+^, Nb^5+^). Moreover, the temperature stability of ceramics mostly depends on the grain boundary activation energy (*E*_gb_) [30], which can be enhanced by element doping too [10,18,19,27,28]. However, there is little research involving manufacturability. During a literature review, we found that TiO_2_, whose *ε*′ decreases with increasing temperature, demonstrating a negative temperature coefficient [31], can simultaneously improve the tan*δ* and temperature stability [7,17,18,19,20], which motivated our curiosity to survey whether it benefits the manufacturability of ceramics.

Powders produced through a sol–gel route offer notable benefits, including precise chemical stoichiometry, uniform composition, and heightened reactivity, thanks to the molecular-level blending of liquid raw materials [32,33]. Hence, the sol–gel method was adopted to synthesize pure CCTO ceramic powders [34]. ZrO_2_ and Nb_2_O_5_ pre-doped CCTO with good initial dielectric properties were adopted as the start material [24]. Then, TiO_2_ was incorporated to modify the performance. The influence of TiO_2_ on the phase structure, morphology, dielectric properties, and temperature stability of the CCTO-based ceramics at different sintering temperatures was comprehensively investigated. Furthermore, the underlying mechanisms for the tan*δ*, temperature stability, and repeatability were further examined from the perspectives of *R*_gb_ and *E*_gb_.

## 2. Experimental

### 2.1. Materials and Methods

The raw materials, including Ca(NO_3_)_2_·4H_2_O (99.0%, Aladdin, Shanghai, China), C_16_H_36_O_4_Ti (99.0%, Macklin, Shanghai, China), and Cu(NO_3_)_2_·4H_2_O (99.0%, China National Medicines Corporation Ltd., Beijing, China), were weighed according to the stoichiometry of CCTO. C_6_H_8_O_7_ (99.0%, China National Medicines Corporation Ltd., Beijing, China), Ca(NO_3_)_2_, and Cu(NO_3_)_2_·4H_2_O were put into a beaker, and enough anhydrous ethanol to just allow all of the solid reagents to be dissolved was added. Subsequently, the mixture was heated and stirred in a water bath at 82 °C until full dissolution was attained. Then, C_16_H_36_O_4_Ti was put into the same beaker, thoroughly stirred, and dried for 10 h to form gel. The gel was heated in a Muffle furnace at 800 °C for 7 h to obtain black CCTO precursor powders with a grain size of about 500 nm (illustrated in Figure S1). Then, 2 mol% ZrO_2_ (99.0%, Aladdin, Shanghai, China) and 1 mol% Nb_2_O_5_ (99.0%, China National Medicines Corporation Ltd., Beijing, China) powders with a size of less than 100 nm were pre-added into the pure CCTO powders. After that, 6 wt.% or 8 wt.% TiO_2_ powders (99.0%, Aladdin, Shanghai, China) with a specification of 60 nm were doped, named as 0.06 Ti and 0.08 Ti, respectively. Subsequently, we grinded the powders in a grinding bowl until a homogeneous mixture was obtained. After sieving through a 120-mesh screen, the powders were pressed with uniaxial static pressure at 250 MPa for 2 min to form pellets with a diameter of 10 mm (shown in Figure S2). Finally, the pellets were placed in a Muffle furnace and sintered in air at 1020, 1030, and 1040 °C for a duration of 8 h to obtain the final ceramic samples. To measure the electrical properties, sandpaper was used to polish the surfaces of the samples, and lead-free silver paste (Shanghai Shiyin Electronic Materials Co., Ltd., Shanghai, China) was coated on each polished surface. Then, the samples were annealed at 620 °C for 30 min to form the electrodes. The final ceramics have a diameter of ~9.1 mm and a thickness of ~1.1 mm.

### 2.2. Characterization Methods

The crystal structures of the TiO_2_-doped CCTO ceramics were determined using X-ray diffraction (XRD) (D/MAX IIIB, Rigaku, Tokyo, Japan), scanning from 10 to 90° at 2θ with a scanning speed of 2°/min. The densities of these samples were accurately measured through the Archimedes method. To analyze the morphology, all CCTO ceramic samples were polished and subjected to a 120 min thermal etching treatment at 900 °C and then observed through a scanning electron microscope (SEM) (VEGA3 SBU, Tescan, Brno-Kohoutovice, Czech Republic). To obtain the dielectric properties and impedance spectra, a wide-band dielectric spectrometer (Concept 80, Novolcontrol, Montabaur, Germany) was employed across a frequency spectrum spanning from 10^−1^ to 10^7^ Hz with a Vrms of 0.5 V and at different temperatures (−125 to 200 °C) in a liquid nitrogen environment (illustrated in Figure S3).

## 3. Results

### 3.1. Phase Structure

**Figure 1:**
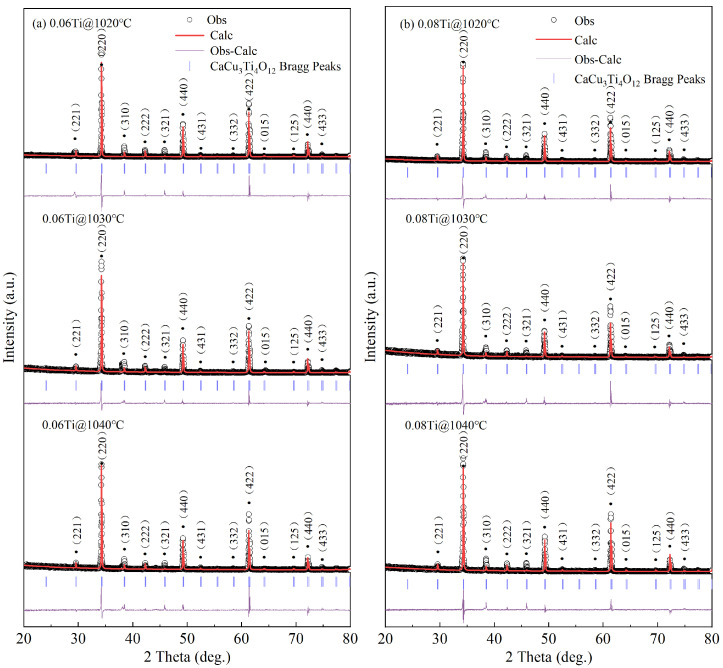
Rietveld refinement and XRD patterns of 0.06 Ti (**a**) and (**b**) 0.08 Ti ceramics sintered at various temperatures.

The XRD patterns for the 0.06 Ti and 0.08 Ti samples sintered at 1020, 1030, and 1040 °C were subjected to Rietveld refinement using Fullprof software (2023, Institut Laue-Langevin, Grenoble, France), and the results are depicted in Figure 1. All peaks exhibit an excellent match with cubic perovskite-related structures (space group Im3). The major phase, CCTO (JCPDS 75-2188), is consistently identified in all sintered samples. In contrast to the CCTO pre-substituted by ZrO_2_ and Nb_2_O_5_ [24], these samples do not exhibit any impurity phase. The absence of an impurity phase can be attributed to the fact that both the radii of Zr^4+^ (0.72 Å) and Nb^5+^ (0.64 Å) are similar to that of Ti^4+^ (0.605 Å), facilitating the substitution of Ti^4+^ in CCTO. Moreover, when the TiO_2_ content in CCTO is below 10 wt%, secondary phases will not be detected in XRD patterns, consistent with previous reports [35,36]. Analysis of the XRD results reveals that the lattice constants (*α*) of CCTO ceramics fall within the range from 7.3980 to 7.3988 Å, surpassing those of pure CCTO ceramics (7.391 Å) [1,37]. The specific *α* values for all samples are detailed in Table 1. This expansion in lattice constants can be attributed to the fact that the doped components may enter the lattice.

### 3.2. Morphology

**Figure 2:**
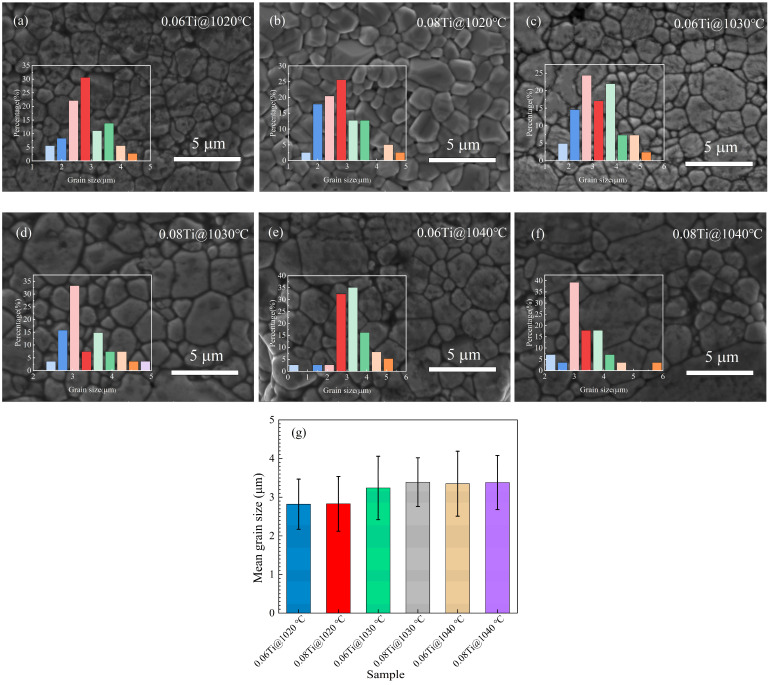
SEM images (**a**–**f**) and mean grain size (**g**) of TiO_2_-doped CCTO-based ceramics sintered at various temperatures.

As one of the important factors affecting the dielectric properties of ceramics, microscopic morphology is of vital significance. Figure 2 shows the SEM images of all of the samples and the average grain sizes estimated by utilizing the linear intercept method. The SEM images in Figure 2a–f exhibit distinct grain boundaries and regular crystal shapes in the samples, each showing low levels of porosity. The average grain sizes for the doped samples fall within the range from 2.82 ± 0.65 to 3.38 ± 0.70 μm (as depicted in Figure 2g, detailed in Table 2) and are less than the that of pure CCTO, which possesses a grain size of 3.53 ± 0.75 μm (illustrated in Figure S4). Specifically, the grain sizes for samples sintered at the same temperature are equal despite the varying TiO_2_ concentration, showing that TiO_2_ concentration has little effect on grain size. At the same time, for samples with identical TiO_2_ concentrations, there is a noticeable trend of increasing grain size with rising sintering temperatures. Table 2 provides the measured density and relative density of 0.06 Ti and 0.08 Ti sintered at different temperatures. The relative density of all samples is about (95.00 ± 1.00)%, indicating the successful preparation of dense CCTO-based ceramics which are almost independent of sintering temperature and TiO_2_ concentration.

### 3.3. Dielectric Performances

**Figure 3:**
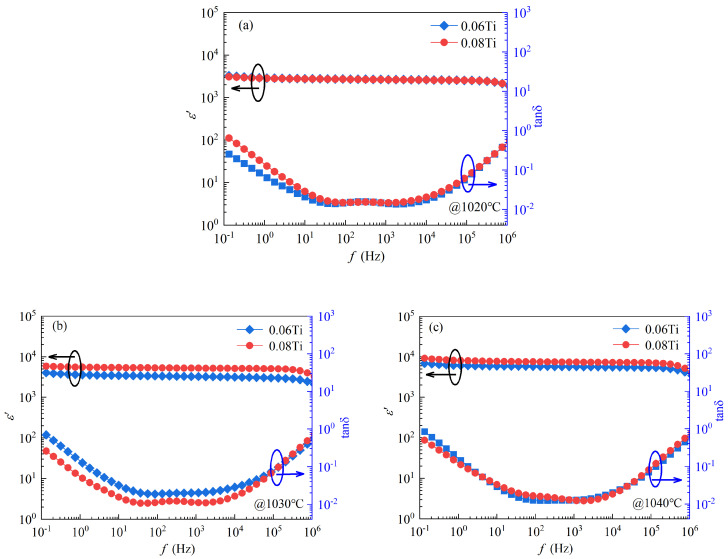
The relationship between the *ε*′ and tan*δ* with respect to frequency for 0.06 Ti and 0.08 Ti samples sintered at 1020 (**a**), 1030 (**b**), and 1040 °C (**c**).

The frequency dependencies between the *ε*′ and tan*δ* for the 0.06 Ti and 0.08 Ti samples sintered at 1020, 1030, and 1040 °C are illustrated in Figure 3. According to Figure 3, in the whole frequency range from 10^−1^ to 10^6^ Hz, the *ε*′ of all samples is high, greater than 3 × 10^3^, and increases with the rise in sintering temperature. Furthermore, all ceramics show nearly unchanged *ε*′ values, indicating excellent frequency stability. A stable *ε*′ is essential for adapting to various working environments, indicating the excellent application potential of the sample.

The tan*δ* is another important dielectric property of CCTO ceramics. The lowest tan*δ* for all samples is about 0.010. Interestingly, across a very wide frequency range from 10 to 10^5^ Hz, the tan*δ* of all samples is below 0.030, which is much lower than that of previous reported CCTO samples [18,30]. What is more, the tan*δ* values are remarkably lower than that of the Zr^4+^ and Nb^5+^ co-substituted CCTO ceramics, whose tan*δ* is 0.23 [24], and also significantly lower than that of pure CCTO, whose tan*δ* is 0.026, verifying again that the addition of TiO_2_ can effectively reduce the tan*δ*. Importantly, sintering at temperatures of 1020, 1030 and 1040 °C, the 0.06 Ti and 0.08 Ti samples present similar tan*δ* values and exhibit good reproducibility.

**Figure 4:**
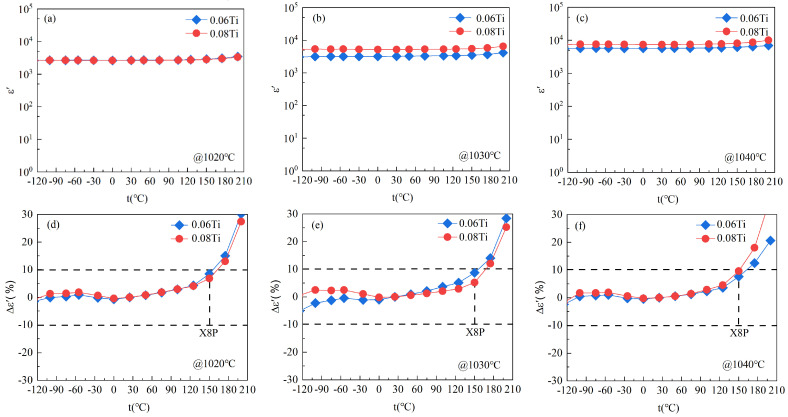
The temperature coefficient line (Δ*ε*′) of the CCTO-based ceramic samples with varying amounts of added TiO_2_ sintered at various temperatures.

Figure 4a–c show the temperature-dependent curves of *ε*′ for all samples. Δε′ was calculated through (*ε*′_T_ − *ε*′_25_)/*ε*′_25_ × 100%, where *ε*′_25_ and *ε*′_T_ represent the *ε*′ at 25 °C and *T*. The functional relationship between Δ*ε*′ and temperature for all samples is shown in Figure 4d–f. All of the *ε*′ values are stable *ε*′ in the temperature range from −125 to 150 °C and tend to increase with higher measuring temperature. The TiO_2_ content and sintered temperature have little impact on the trend of *ε*′. Within the temperature range from −125 to 150 °C, both 0.06 Ti and 0.08 Ti at the three sintering temperatures exhibit Δ*ε*′ values within ±10%, meeting the standards of the X8P capacitor [38]. Notably, 0.08 Ti sintered at 1020 and 1030 °C demonstrated a Δ*ε*′ close to ±5%, indicating excellent temperature stability. According to previous research results [31], this phenomenon may be ascribed to the existence of the negative temperature coefficient of TiO_2_ in the samples. These doped ceramics with different TiO_2_ concentrations sintered at temperatures of 1020, 1030, and 1040 °C display equal and excellent temperature stability, demonstrating good reproducibility.

## 4. Discussion

**Figure 5:**
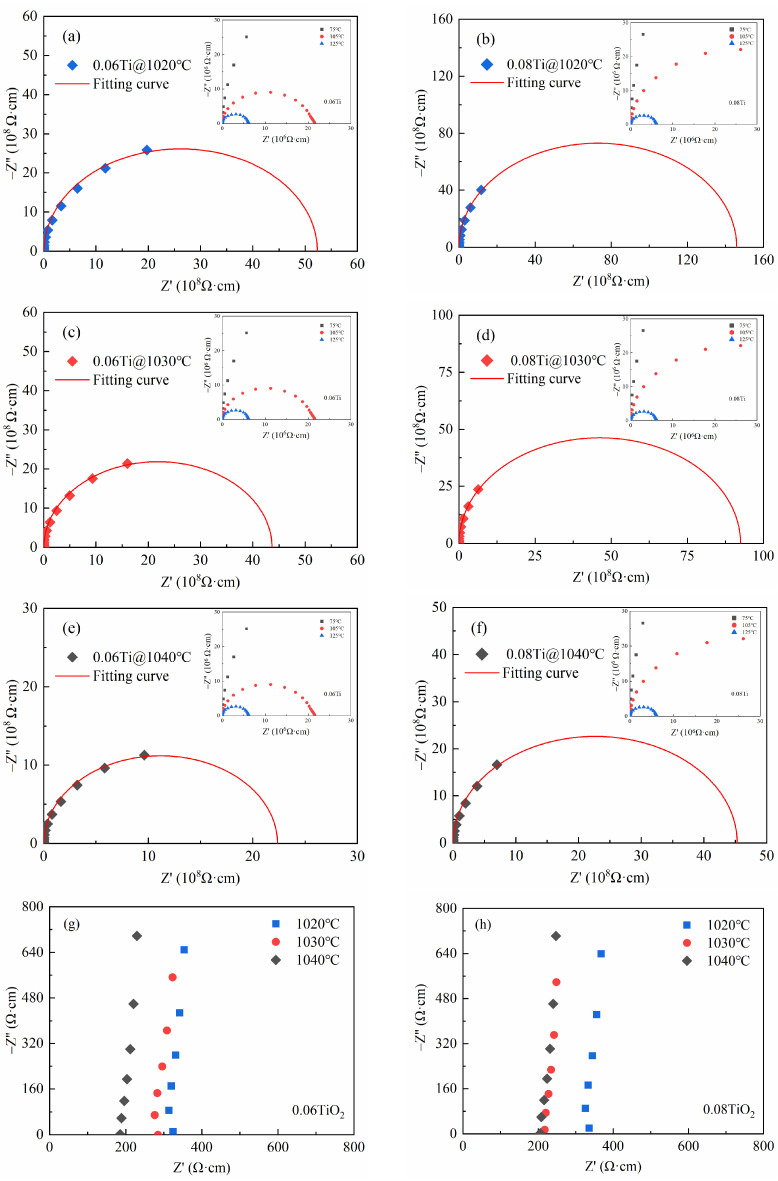
Complex impedance spectra (Z*) fitted at room temperature for 0.06 Ti and 0.08 Ti (**a**–**f**); insets show Z* at high temperatures (75, 105, and 125 °C); enlarged view of high-frequency region of 0.06 Ti (**g**) and 0.08 Ti (**h**).

To investigate the mechanism behind the enhanced temperature stability of *ε*′ in these prepared ceramics due to the addition of TiO_2_, we utilized complex impedance analysis to reveal the impact of TiO_2_ addition on *R*_g_ and *R*_gb_. According to the internal barrier layer capacitance model, which is the most accepted explanation for the high permittivity in polycrystalline CCTO ceramic at present, complex impedance spectroscopy stands out as a potent method for unveiling the electrical heterogeneity in CCTO ceramics [39]. The impedance can be elucidated through a series-connected resistor–capacitor parallel circuit, and its equivalent circuit is widely employed in the investigation of the grains’ and grain boundaries’ electrical response in CCTO-based ceramics [40,41]. The following equation is used for calculating the complex impedance [42]:

(1) $$Z^{*}=Z^{\prime }-jZ^{\prime \prime }=\frac{R_{\mathrm{gb}}}{1+j\omega R_{\mathrm{gb}}C_{\mathrm{gb}}}+\frac{R_{g}}{1+j\omega R_{g}C_{g}},$$

where $Z^{*}=Z^{\prime }-iZ^{\prime \prime }$ is the complex impedance, ω = 2π*f* represents the angular frequency, and $C=\varepsilon_{0}A/d\;$ is the vacuum capacitance. Typically, the values of *R*_gb_ and *R*_g_ are ascertainable through the observation of a prominent semicircular arc at lower frequencies and a non-zero intercept on the *Z*′ axis at higher frequencies in the Z* plane plots, respectively [43]. Figure 5 plots the complex impedance spectra for 0.06 Ti and 0.08 Ti at room temperature, 75, 105, and 125 °C. From Figure 5a–f, it can be observed that due to limitations in the measurement frequency, only a large semicircular arc is visible for the spectra at room temperature. Hence, precise values for *R*_g_ and *R*_gb_ can be derived through the fitting of the measured complex impedance data. After fitting, the *R*_gb_ for 0.06 Ti ranges from 2.48 × 10^9^ to 6.00 × 10^9^ Ω, which is significantly greater than *R*_g_ (ranging from 185.5 to 324.5 Ω). Meanwhile, the *R*_gb_ for 0.08 Ti is in the range from 5.68 × 10^9^ to 10.83 × 10^9^ Ω, also far exceeding *R*_g_ (ranging from 206.1 to 335.6 Ω), demonstrating pronounced electrical heterogeneity [44]. These *R*_gb_ values exceed the previously reported value of 1.46 × 10^9^ Ω for pure CCTO ceramics [30]. The higher *R*_gb_ is a key factor contributing to the low tan*δ* observed.

**Figure 6:**
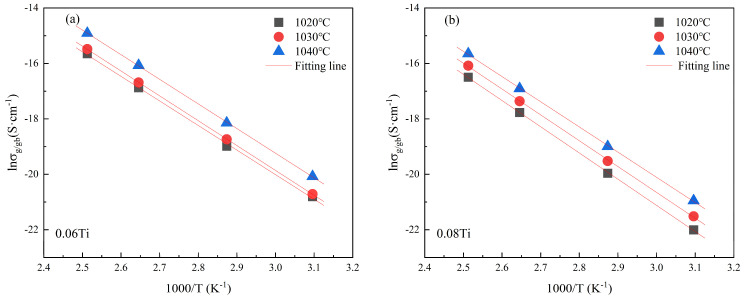
Arrhenius plots of ln*σ*_gb_-1000/T for 0.06 Ti (**a**) and 0.08 Ti (**b**) sintered at different temperatures.

Both grain conductivity (*σ*_g_) and grain boundary conductivity (*σ*_gb_) adhere to the Arrhenius law as follows [30]:

(2) $$\ln\sigma_{\mathrm{gb}}=(\frac{-E_{\mathrm{gb}}}{k_{B}T})+\ln\sigma_{0},$$

where *E*_gb_ and *σ*_0_ represent the grain boundary activation energy and the pre-exponential factor, respectively. *K*_B_ and *T* are the Boltzmann constant and the absolute temperature. The determination of *σ*_gb_ values in CCTO ceramics involves the calculation based on complex impedance spectra data collected at different temperatures. Upon adherence to the Arrhenius law of the experimental data, the *E*_gb_ values can be obtained from the slopes of the fitting lines. From the fitting lines in Figure 6a,b, the *E*_gb_ values for the 0.06 Ti samples sintered at 1020, 1030, and 1040 °C were 0.76, 0.77, and 0.77 eV, respectively. Similarly, the *E*_gb_ values for the 0.08 Ti samples sintered at these temperatures were 0.78, 0.81, and 0.80 eV. Those *E*_gb_ values are notably higher than the reported results earlier (range of 0.63–0.72 eV) [18,22,30]. The temperature stability and the similar performance of this series of samples may possibly be attributable to their large *E*_gb_ values. These results manifest that TiO_2_ doping is capable of effectively increasing the value of *E*_gb_, thereby enhancing the temperature stability of CCTO products.

Through the above analyses, it can be found that the TiO_2_-added CCTO ceramics show more uniform performance, including a similar tanδ and equal temperature stability, and less sensitivity to TiO_2_ content and sintering temperature, which can raise the yield and lower the requirement for homogeneity in the sintering temperature, reducing the difficulty in manufacture, facilitating manufacturability, and demonstrating the significant influence of TiO_2_ doping on the reproducibility and the manufacturability of CCTO ceramics. The mechanism may be related to the good temperature stability of TiO_2_ and its insensitivity to sintering temperature change, which is worthy of further study in the application of CCTO ceramics. Additionally, different from previous fabrication routes in other papers [7,17,18,19,20], such as solid oxide methods or polymer pyrolysis, the special preparation of “CCTO powders via the sol–gel method followed by TiO_2_ particle addition”, which can allow most of the added TiO_2_ to possibly accumulate in the grain boundaries, may be responsible for the high R_gb_ and E_gb_ and therefore the high performance.

## 5. Conclusions

This study investigates the influence of TiO_2_ doping on the tan*δ,* temperature stability, and repeatability of CCTO-based ceramics prepared using the sol–gel method. The ceramics co-doped with TiO_2_ and sintered at temperatures of 1020, 1030, and 1040 °C exhibited X8P temperature stability and a low tan*δ,* consistently below 0.03 within a frequency range from 10 to 10^5^ Hz, suggesting that they are promising candidates for dielectric materials in capacitors. Moreover, the similar characteristics also prove that TiO_2_ is a powerful additive for mitigating the performance difference induced by sintering temperature, thereby reducing the strict requirements in weighing TiO_2_ additives and the uniformity of sintering temperature, which facilitates the industrialization of power capacitors based on CCTO ceramics that are featured with poor repeatability.

## Figures and Tables

**Table 1:** Rietveld parameters of 0.06 Ti and 0.08 Ti at various temperatures.

	0.06 Ti @1020 °C	0.08 Ti @1020 °C	0.06 Ti @1030 °C	0.08 Ti @1030 °C	0.06 Ti @1040 °C	0.08 Ti @1040 °C
Profile function	Pseudo Voigt	Pseudo Voigt	Pseudo Voigt	Pseudo Voigt	Pseudo Voigt	Pseudo Voigt
Crystal system	Im3	Im3	Im3	Im3	Im3	Im3
Lattice parameters a = b = c (Å)	7.3980	7.3984	7.3987	7.3988	7.3983	7.3988
Angle (in degree)	90	90	90	90	90	90
Volume (Å^3^)	404.900	404.961	405.014	405.022	404.944	405.020
Chi-Square value (Chi^2^)	6.44	4.28	4.18	2.79	3.39	2.48
Theoretical density (g/cm^3^)	5.038	5.038	5.036	5.037	5.037	5.053

**Table 2:** Tan*δ* (min), measured density, relative density, and mean grain size of 0.06 Ti and 0.08 Ti sintered at various temperatures.

Sample	tan*δ* (min)	Measured Density (g/cm^3^)	Relative Density (%)	Mean Grain Size (μm)
0.06 Ti@1020 °C	0.014	4.817	95.61	2.82 ± 0.65
0.08 Ti@1020 °C	0.013	4.798	95.24	2.83 ± 0.71
0.06 Ti@1030 °C	0.010	4.834	95.99	3.24 ± 0.82
0.08 Ti@1030 °C	0.012	4.833	95.95	3.38 ± 0.63
0.06 Ti@1040 °C	0.012	4.791	95.12	3.35 ± 0.84
0.08 Ti@1040 °C	0.012	4.771	94.42	3.38 ± 0.70

## Data Availability

The raw data supporting the conclusions of this article will be made available by the authors on request.

## Supplementary Materials

The following supporting information can be downloaded at: https://www.mdpi.com/article/10.3390/ma17235824/s1, Figure S1: SEM images of pure CCTO powder obtained via sol–gel route; Figure S2: SEM images of cross-section of 0.06Ti (a) and 0.08Ti (b) ceramic plates before sintering; Figure S3: The sample cell electrodes (a); the placement diagram of the sample and additional external electrodes (b); Figure S4: SEM image of pure CCTO ceramic after sintering at 1200 °C for 8 h.
