# Supplementary material for: The Effect of TiO2 on the Dielectric Performance of ZrO2 and Nb2O5 Pre-Doped CaCu3Ti4O12 Ceramics
Source: Materials (Basel). 2024 Nov 27;17(23):5824. doi: 10.3390/ma17235824 (PMC11642029; doi:10.3390/ma17235824)
Supplement: Supplementary file 1 [file materials-17-05824-s001.zip › materials-3299817-supplementary.pdf]

Supplementary Materials

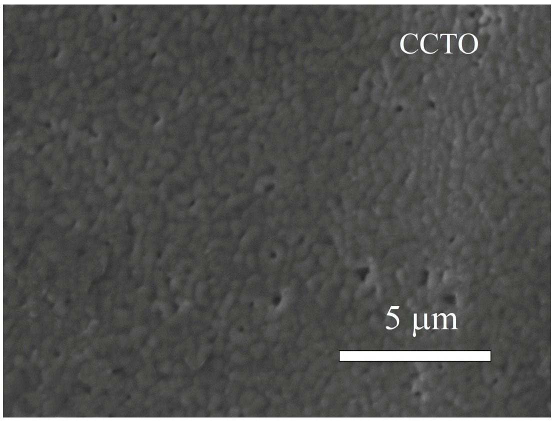

Figure S1. SEM images of pure CCTO powder via sol-gel route.

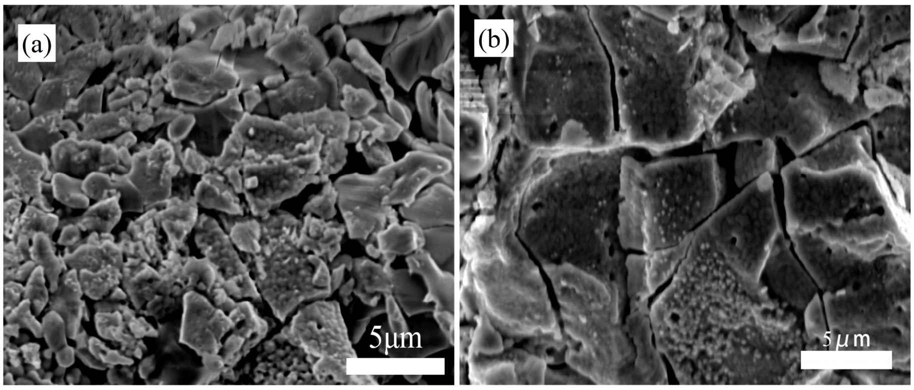

Figure S2. SEM images of cross section for 0.06Ti (a) and 0.08Ti (b) ceramic plates before sintering.

Please note the every particle is not crystalline grain and includes many very small powders.

(a)

(b)

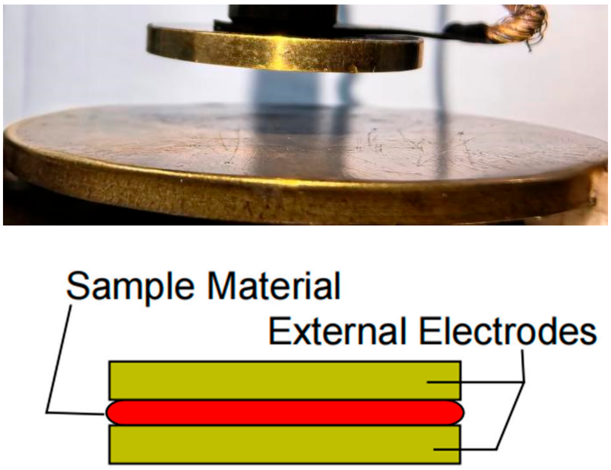

Figure S3. The sample cell electrodes (a), the placement diagram of sample and additional external electrodes (b).

When conducting the measurement, the sample is not directly inserted into the sample cell electrodes. Instead, the sample coated with Ag was prepared in sandwich structure between additional external electrodes as shown above.

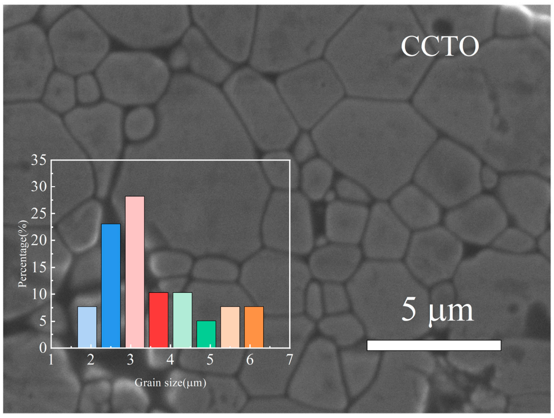

Figure S4. SEM image of pure CCTO ceramic after sintering at 1200 °C for 8 h.
